# 24 h-Heart Rate Variability as a Communication Tool for a Personalized Psychosomatic Consultation in Occupational Health

**DOI:** 10.3389/fnins.2021.600865

**Published:** 2021-02-11

**Authors:** Marc N. Jarczok, Thomas Buckley, Harald O. Guendel, Irina Boeckelmann, Daniel Mauss, Julian F. Thayer, Elisabeth M. Balint

**Affiliations:** ^1^Clinic for Psychosomatic Medicine and Psychotherapy, University Hospital Ulm, Ulm, Germany; ^2^Faculty of Medicine and Health, The University of Sydney, Sydney, NSW, Australia; ^3^Leadership Personal Center Ulm (LPCU), University of Ulm, Ulm, Germany; ^4^Occupational Medicine, Faculty of Medicine, Otto-von-Guericke University, Magdeburg, Germany; ^5^Mannheim Institute of Public Health, Social and Preventive Medicine, Medical Faculty Mannheim, Heidelberg University, Heidelberg, Germany; ^6^Department of Psychological Science, School of Social Ecology, University of California, Irvine, Irvine, CA, United States

**Keywords:** 24 h-HRV, FFT, consultation, occupational health, workplace, screening

## Abstract

New tools for non-specific primary prevention strategies covering somatic and mental health in occupational medicine are urgently needed. Heart rate variability (HRV) reflects the capacity of the body to adapt to environmental challenges and of the mind to regulate emotions. Hence, a 24 h-measurement of HRV offers a unique possibility to quantify the interaction between situation-specific emotional regulation within a specific psychosocial environment and physiological state, thereby increasing self-perception and inducing motivation to change behavior. The focus of the present study represents such a 24 h-measurement of HRV and its presentation as a comprehensive graph including protocol situations of the client. A special training program for occupational health physicians and questionnaires for clients were developed and administered. The article reports the first data of the study “*healthy leadership and work – body signals for managers and employees*”, an investigator-initiated, interventional, single-arm, open (non-blinded), multicenter, national trial with 168 participants. They reported a significantly improved perception of their bodily needs after the consultation (from Median = 7, interquartile range 5–8 to Median = 8, interquartile range 7–9; scale range from 1 to 10; *p* < 0.001, Wilcoxon rank test; effect size 0.49). The 16 occupational health physicians stated that the measurement of HRV was very well suited to enter into dialog with the managers and was feasible to show interactions between situations, thoughts, feelings, and bodily reactions. Taken together, we show that a 24 h-HRV-measurement can be a feasible and effective approach for holistic, psychosomatic primary prevention in occupational medicine. We discuss possible mechanisms for improving the individual health via the consultation, containing mindset and improved ANS activity.

## Introduction

The number of people with chronic diseases is increasing in society, for example, affecting approximately 40% of all Germans (Robert Koch-Institut (Hrsg.), 2014). The WHO attributes 60% of all deaths worldwide to chronic diseases, most of them to cardiovascular diseases (CVD) in Western countries (World Health Organisation, 2005). The costs of illness caused by CVD amounted to 46.4 billion Euros in Germany in 2015, whilst only 0.3% of this was spent on prevention in 2017 (DeStatis (Hrsg.), 2017; OECD (Hrsg.), 2020). Although CVD risk is partially determined by genetic factors, lifestyle plays an important preventative role; for example, physical activity and sleep can be associated with a reduced CVD risk of approximately 50% (Odegaard et al., 2011). Hence, opportunities for preventive activities are urgently needed. The present study investigates such an opportunity by implementing a 24 h-heart rate variability (HRV)-based consultation in a health check-up at the workplace, as this is a promising place for preventive activities.

Preventive activities at the workplace have the potential to address a large proportion of the population aged 20–64 as in Europe, the employment-to-population-ratio is 73%, meaning that 3 of 4 people interact with a workplace (Eurostat (Hrsg.), 2020). Thus, practical tools to A) easily assess an individuals current health state, B) increase knowledge about one’s risks and resources, and improve understanding of bodily needs, and C) increase the motivation to change behavior are of particular interest in this area.

Nonetheless, existing preventive medical check-ups in occupational medicine often focus on secondary prevention only, as they merely detect an existing disease at an early stage or estimate the risk for a specific disease. For example, the *Findrisk* score assesses the risk of developing type 2 diabetes (Lindström and Tuomilehto, 2003) and the Framingham risk score estimates the cardiovascular (CV) risk within the next 10 years (Wilson et al., 1998). The risk of developing type 2 diabetes or CV diseases (CVD) is very low at younger ages, a time when primary prevention strategies should rather be implemented. On the other hand, addressing behavioral components of primary prevention such as engaging in physical activity, dietary habits, or stress prevention is not specific to a single disease. Trying to measure the effects of more general aspects like work stress raises several questions. Most commonly, the assessment of work stress is limited by missing objectivity as it is predominantly based on individuals’ subjective perception and hence, self-report only (i.e., questionnaires). Although the association of perceived stress levels with various diseases has been reported (Backé et al., 2012; Lundberg, 2015; Theorell et al., 2015), the assessment of stress remains problematic at an individual level. The link between stress and disease risk is confounded by different variables such as individual genetic predisposition, sex, early life experience, individual resources, and actual coping strategies (Schneiderman et al., 2005; Rückholdt et al., 2019).

Unfortunately, most objective biological methods to measure stress levels are expensive, invasive, and often complicated to perform. For example, assessing a full cortisol circadian profile (a prominent stress hormone) comprising eight saliva samples is accompanied by low compliance because many participants perceive the strict protocol (exact timings, no food, no coffee, no cigarettes in the first hour post awakening) as impractical and protocol violation results in poor reliability (Stalder et al., 2016).

In addition to the hypothalamic-pituitary-adrenal (HPA) axis and associated cortisol response, there is another important stress pathway involving the central autonomic network (CAN) that is relaying its information primarily through the autonomic nervous system (ANS). This pathway can be indexed by measuring the variability between heartbeats, a valid measure of autonomic function (Wulsin et al., 2018). Two major components of the ANS can be differentiated. The parasympathetic component includes the cardiac branch of the 10th cranial nerve (i.e., the vagus nerve), which tonically inhibits intrinsic heart rate and modulates it on a beat to beat basis in milliseconds. In contrast, the sympathetic component modulates the heart rate at a slower frequency on a magnitude of seconds (below about 0.15 Hz). In detail, the vagus nerve represents a primary, bidirectional, and fast route transmitting physiological state to the brain (sensory part) as well as modulating somatic responses (motor part) to adapt to environmental challenges such as work demands (Bernard, 1867; Thayer and Lane, 2000; Wulsin et al., 2018). At the brain’s end, the CAN controls the activity of preganglionic sympathetic and parasympathetic neurons. The CAN is involved in not only moment-to-moment modulation of visceral functions such as heart rate as described above but also in adaptation to internal or external challenges and the maintenance of homeostasis (Benarroch, 2014). Its network is organized on multiple levels, of which three are particularly important to this work: the forebrain – including the insular cortex, and anterior cingulate cortex, the limbic system including the amygdala, and the hypothalamus. Notably, these first three regions are involved in the integration of bodily sensations with emotional and goal-related autonomic responses, while the hypothalamus controls the integration of endocrine, autonomic, and sleep responses for homeostasis and adaptation. The second level includes, among others, the periaqueductal gray which integrates autonomic control of pain modulation and sleep, and the behavioral responses to environmental challenges (i.e., often termed stress) (Benarroch, 2014). Yet, it is the appraisal that turns an environmental or internal stimulus into a biological relevant stressor (Folkman et al., 1986). Thus, cardiac variability measures can provide a window to the working level of the CAN that reflects the capacity of the body to adapt to environmental challenges (Thayer et al., 2011) and to regulate emotions (Thayer and Lane, 2000), both pivotal in handling, e.g., work stress, like a problematic supervisor or deadline(s). The extent of the variability of heart rate is often used to predict intermediate or terminal endpoints (morbidity endpoints) such as the onset of depression or CVD, and mortality risk, such as from all causes, cardiovascular (e.g., hypertension), and cancer. For example, decreased values of HRV predict premature mortality and morbidity, e.g., higher inflammatory state (Jarczok et al., 2014; Aeschbacher et al., 2017), increased CVD risk (Kristal-Boneh et al., 1995; Mercedes et al., 2002; Jarczok et al., 2013b; Schuster et al., 2016) and myocardial infarction (MI) risk (Thayer and Lane, 2007). Research has also reported associations with common mental disorders like depression (Kemp et al., 2014). Apart from diagnosed diseases, HRV parameters show associations to subjective measures like work stress (Jarczok et al., 2013a, 2020) and self-rated health (Jarczok et al., 2015). Results from five systematic reviews (partly overlapping) related to different aspects and years of occupational stress research suggest that various stress models such as the Job-Demand-Control Model (Karasek, 1979), the Effort-Reward-Imbalance Model (Siegrist, 1996), the concept of Organizational (In)justice (Elovainio et al., 2006; Herr et al., 2012), the perceived stress scale (Cohen et al., 1983) and burnout risk (MBI) (Maslach and Jackson, 1981) were found in the majority of studies to be associated with lower values of HRV measures. Specifically, higher questionnaire scores representing increased strain are associated with reduced parasympathetic activation (decreases in RMSSD and HF-power, but also LF-power and total power) (Togo and Takahashi, 2009; Jarczok et al., 2013a, 2020; Järvelin-Pasanen et al., 2019). In addition, lower SDNN values show a significant correlation with higher mortality in patients with prior MI in large cohort studies (Chattipakorn et al., 2007; Buccelletti et al., 2009; Huikuri and Stein, 2013), bypass surgeries (Lakusic et al., 2013) or heart failure (Sandercock and Brodie, 2006).

Beyond reported correlations between reduced short-time-measures of HRV and disease, measures of HRV capture a well-defined multi-phase course during progressively increasing physical exertion, under standardized working conditions, and during recovery after varying degrees of exertion (Kaikkonen et al., 2010). Thus, HRV can be used as a process-integrated measurement for an objective view of the response to the workload over the working day. Based on this measurement, working conditions can be analyzed for the identification of problematic patterns in the work environment (Bläsing, 2017).

Given these findings, the measurement of HRV is already used in various areas of occupational medicine and occupational science. This includes the analysis of the individual physical and mental workload and the identification of core areas of work-related stress, determination of recovery behavior, evaluation of the impact of new work equipment and new technologies, and application for the risk stratification of CVD (Sammito et al., 2015). Currently, HRV measurement is used for assessing stress and strain elicited by the introduction and usage of new work equipment and new technologies such as digital assistance systems, head-mounted displays, or exoskeleton (Schwerdtfeger et al., 2009) and in Space Medicine to describe the demands on flight personnel and to monitor individuals working under extreme conditions (Baevskiĭ, 2002).

Another example of a feasible application of HRV analysis to quantify 24-h stress/recovery balance in the workplace setting is the use of Firstbeat Bodyguard 2 (BG2) at StriveStronger, Sydney, an executive wellbeing and performance laboratory. The report is integrated into a physiology review consultation creating an opportunity to discuss the impact of lifestyle and health behaviors on stress and recovery (autonomic) balance and is used as an objective method to measure and quantify the effect of behavior change over time. This technology has been successfully used in over 1,000 clients and has been well-tolerated and acceptable as part of executive human performance and physiology assessments (May and Buckley, 2019). Taken together, ANS activity can index the overall functioning and adaptability of the body and mind (Holzman and Bridgett, 2017). Following from the writings of Darwin, in which the bi-directional communication between the heart and the brain was enunciated, the neurovisceral integration model has sought to further explicate this connection using modern methodologies and conceptions (Thayer et al., 2011).

Therefore, measuring ANS activity as a non-specific test would be a desirable instrument in primary prevention, as it allows on the one hand to assess the cumulative interaction of individuals with their environments and on the other hand capture the effect of prevention strategies. Taken together, measures of HRV fulfill many criteria of an early, non-specific screening tool in primary prevention in the area of occupational medicine.

Yet, using a short-term measurement of HRV (<1 h), it is only possible to give the client feedback on his current condition in the sense of a risk assessment such as blood pressure or cholesterol level. A 24 h measurement allows analysis of the course of the work load as described above. We state that far beyond the purely physical load, it represents a holistic assessment of the psychobiological interaction and functioning. Specifically, if the client writes a diary of memorable or important situations while using a wearable device that records HR, an offline analysis approach can be realized. This HR time series can be processed using spectral methods to show to the client a single graph, that contains the variability across the day paired with protocol situations of the client (Jarczok et al., 2019). The graph demonstrates the impact of situations relevant to the client on the visualized time series. This more holistic consultation has great potential to reveal the individual situation-specific psychophysiological reaction, thereby increasing self-perception and, even more importantly, induces a motivation to change behavior.

In particular, this novel approach offers the unique possibility to visualize the interaction between situation-specific emotion regulation (feelings and thoughts) within a specific psychosocial environment and the resulting situation and emotion-specific physiological states - actually the core definition of psychosomatics. The graph (spectrogram) serves as a “door opener” for the consulting clinician and allows to demonstrate individual somatic reactions in specific situations, preparing the ground for changes in attitudes and behavior. For example, a manager thought that he shows positive, healthy behavior by not reading any business emails before bedtime, only non-business ones. However, the HRV graph demonstrated to him that his physiological state was not different between private and business emails. As a result, he changed his bedtime routine and thus improved his sleep quality, which was confirmed in a follow-up HRV assessment. More generally spoken, the clients’ benefit consists of mentalization and reflection of daily routine and corresponding physiological states, showing the individual “wear and tear” of the body, as Seeman would term it (Seeman et al., 1997). Furthermore, not only risky behaviors, but also resources are detected, so that not only negative behaviors are identified, but existing positive behaviors are encouraged. This personalized and salutogenic approach goes far beyond general advice and can create more credibility, insight, and motivation for change. This approach could be seen as next-generation, psychosomatic biomonitoring that covers somatic and mental health in public health settings, empowering clients to take better care of themselves by improving their understanding of how their body and mind are working and where their risks and resources are.

The present article will describe the implementation of a 24 h-HRV-based consultation in a health check-up at the workplace and show the first results. We developed special training for occupational health physicians (OHPs) to enable them to record, analyze, interpret, and discuss these 24 h-measurements of HRV in a “psychosomatic way” (as described above) with the participants. We hypothesize that the HRV-based consultation improves the psychosomatic comprehension of the managers and employees as indicated by a significant increase in their self-reported notice of body signals and that the training is feasible, i.e., enables the OHPs to perform the HRV measurement, interpretation, and consultation.

## Materials and Methods

### Methods

The study “*healthy leadership and work – body signals for managers and employees*” represents an investigator-initiated, interventional, single-arm, open (non-blinded), multicenter, national trial. It is registered in the German Clinical Trials Register (ID: *DRKS00014653*) and approved by the local ethics committee (ID *188/18*, IRB of Ulm University, Germany). Enrollment started on 04/07/2018 at four study sites: an automotive company (A), a multinational engineering and technology company (B), a printing group (C), and a pharmaceutical and consumer goods manufacturer (D). Consultation and data collection were completed at study sites A and B, and ongoing at sites C and D. Therefore, we report complete data from study sites A and B and partial data from the OHPs of study sites C and D.

### Psychometric Data

#### Psychosomatic Interactions

Attitudes to and perceptions of psychosomatic interactions were assessed via eight questions using a Likert scale between 1 and 10 (see Figure 3 for the exact wording).

Satisfaction with the consultation was assessed by 11 questions that were answered on a Likert Scale between 1 and 10 (see Figure 2 for the exact wording).

#### Ability to Work

Workability was assessed by the single question: “If you rate your best workability ever achieved with 10 points: How many points would you give for your current ability to work?” with potential responses between 0 (totally unfit for work) and 10 (currently the best working capacity) (Airaksinen et al., 2018).

#### Irritation Scale

The irritation scale (Mohr et al., 2005) has a hierarchical structure with the two primary factors: cognitive irritation in the sense of not being able to switch off (indicated by items 1, 2, 4) and emotional irritation in the sense of an agitated irritation (items 3, 5, 6, 7, 8). Higher scores indicate higher irritation.

#### Patient Health Questionnaire (PHQ-4)

The 4-item Patient Health Questionnaire (Löwe et al., 2010) consists of four items, two measuring anxiety, two depression, that are scored between 0 = never and 3 = almost every day and are summed, yielding a sum score between 0 and 12. Internal reliability Cronbach’s Alpha previously reported for this questionnaire is 0.82 (Löwe et al., 2010). Higher values indicate more anxious and depressive symptoms. Sum scores of ≥3 in each subscale are categorized as probable cases of depression or anxiety, respectively (Löwe et al., 2005; Kroenke et al., 2009).

#### Perceived Stress Scale (PSS-4)

Four questions ask participants how often they experienced stressful situations in the previous month on a Likert scale between 0 = never and 4 = very often (Cohen et al., 1983; Klein et al., 2016). Higher values indicate more stress. Cronbach’s Alpha reported for this scale is 0.77 (Warttig et al., 2013).

### Details Regarding the HRV Measurement

A single-channel-ECG (Sampling rate 1000 Hz) was recorded using either three electrodes (Ambu BlueSensorR ECG Electrodes REF R-00-S/25) and a cable set (Bittium Corp., Oulu, Finland) or a textile chest belt with dry electrodes and the corresponding stingray adapter (Bittium Corp., Oulu, Finland) for at least 24 h using Bittium Faros^TM^ 180 (Bittium Corp., Oulu, Finland). The ECG was imported into HRV-Scanner Software (BioSign GmbH, Ottenhofen, Germany) at study site A and into CardiscopeTM ANALYTICS professional edition (HASIBA medical GmbH, Graz, Austria) at study site B, screened and edited for artifacts and HRV values were calculated for consultation. The scientific HRV analysis was conducted by EB using the CardiscopeTM ANALYTICS professional edition (HASIBA medical GmbH, Graz, Austria). Only ECGs with a minimum recording length of 22 h (79,200 s) were entered into the analyses. The automated recognition of regular rhythm and artifacts of the software was checked manually and ECGs entered statistical analyses only if the rate of sinus rhythm was higher than 90%, regardless of the cause (aberrant rhythms or artifacts). The following parameters were extracted for analyses: HR (mean for 24 h), SDNN (standard deviation of all RR intervals), SDNN-i (mean value of the standard deviations of the average RR intervals of all 5-min segments of a measurement), SDANN (standard deviation of the average RR intervals of all 5-min segments of a measurement), RMSSD (square root of the squared mean of the sum of all differences of successive RR-intervals), Total Power-i (average power density in the total band, i.e., between 0.0 and 0.4 Hz of all 5-min-calculation windows), HFi (average energy density in the HF (high frequency) band, i.e., between 0.15 and 0.4 Hz of all 5-min-calculation windows), LFi (average energy density in the LF (low frequency) band, i.e., between 0.04 and 0.15 Hz of all 5-min-calculation windows). For a description of the HRV parameters see Shaffer and Ginsberg (2017) and for further information of 24 h-analysis see Jarczok et al. (2019).

### Survey of the OHPs

The authors EB and MNJ met OHPs regularly (3, 6, and 12 months after the initial training) to review progress and address any questions. At each meeting, a standardized question catalog regarding difficulties in presenting the project, handling the devices, questionnaires and software, evaluating and interpreting the HRV measurement and conducting the consultations (see Supplementary Table 1 for the exact wording) was discussed. The answers of the OHPs were recorded verbatim and summarized for this article. Further, the OHPs completed a questionnaire directly after the training with questions regarding the perceived quality of the training, scaled on a Likert Scale between 1 and 6, and a questionnaire at the end of the project (12 months after the initial trainings) asking for their opinion regarding the effort for explaining, analyzing, interpreting, discussing the measurement, how helpful it was for the consultation, whether and in which context they wanted to continue using the HRV measurement and questions about the training and meetings. All questions were answered on a Likert Scale between 1 and 10.

### Study Plan

We recruited OHPs through personal contact. The complete team of OHPs received training with a 1-day workshop to teach basic knowledge of HRV, details of measurement, analysis, and interpretation, including a hands-on part with clinical cases and ECG data (case vignettes) (see Supplementary Table 1). After practicing with some example cases with individual feedback from the supervisors EB and MNJ, the OHPs offered the HRV measurement and consultation at their company. Individual supervision for the OHPs was provided by study investigators (EMB) in case of need. Three, 6, and 12 months after the initial training, meetings were organized where the OHPs presented actual cases.

In study site A, the measurement was offered to every manager who consulted the OHP for his regular health check-up. In sites B–D, HRV-based consultation was offered not only to managers but also to employees by written information available at the company’s medical service, not associated with a certain check-up.

Following written consent, participants received a 24 h recording of ECG and they were asked to complete a diary of that day and a questionnaire. The latter contained standard demographical questions, questions about hours actually worked weekly, smoking, hours of sports/physical activity per week, knowledge of relaxation methods like yoga, progressive muscle relaxation, autogenic training (yes/no), practicing a relaxation method, a question regarding subjective sleep quality [“*How do you rate your sleep overall?*” Scale from 1 (excellent) to 5 (poor)], perceived psychosomatic interactions [e.g. “*Feelings are expressed in me. – mentally (1) – physically (10)*”], workability, and the following standardized questionnaires: irritation scale (IS), perceived health questionnaire (PHQ-4) and perceived stress scale (PSS-4). Afterward, the OHP analyzed the ECG with regard to HRV and discussed the results with the study participant during their next meeting. Immediately after the consultation, the participant was asked to complete a questionnaire regarding his satisfaction with the consultation and questions about perceived psychosomatic interactions again. OHPs recorded medical diagnoses, medication, diastolic, and systolic blood pressure (only study site A), height, and weight (only study site A). CV risk factors comprised: smoking, arterial hypertension, hypercholesterinemia, adiposity (BMI ≥ 30 kg/m^2^), diabetes (Type I or II), and hyperuricemia. At study site B, OHPs recorded the reason for the measurement (request of employee/manager, suggestion of OHP with explanation). Data were collected between July 2018 and June 2019 for 12 months at study site A, between December 2018 and August 2020 at site B, between May 2019 and ongoing at sites C and D.

**FIGURE 1:**
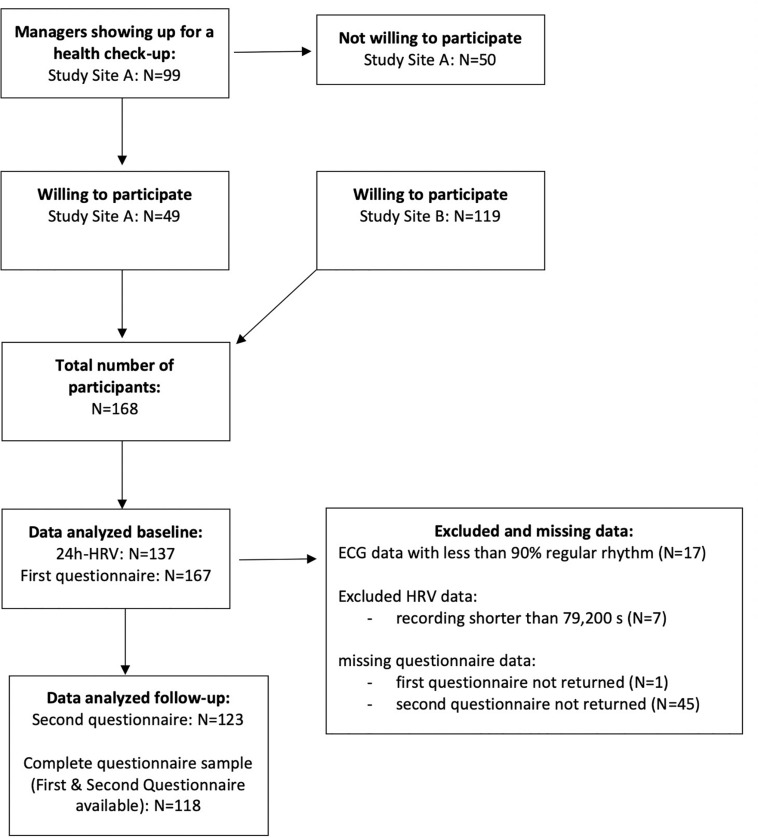
Flowchart of recruitment.

Inclusion criteria for participation were age between 18 and 65 years and being a manager or employee at the cooperating company at the time point of study inclusion. Exclusion criteria were history of permanent cardiac arrhythmias (atrial fibrillation), pacemaker-dependent rhythm, or conditions under which calculation of HRV is not possible (e.g., frequent extrasystoles). The flowchart of the recruitment is shown in Figure 1.

The primary outcome was an increase in the self-reported notice of body signals in employees and managers pre/post via a question in the questionnaire: *“I can easily perceive what my body needs*… *Do not agree at all – strongly agree*” (scale from 1 to 10). This question was asked before the HRV measurement and immediately after the consultation.

### Details of HRV Analysis and Consultation

After importing the ECG data into the HRV software, the quality of data was reviewed by checking the parameters presented by the software (percentage of recognized regular rhythm) and by checking the ECG in time frames in which the software marked less recognized regular rhythm. For a valid interpretation of HRV, the artifact rates should be lower than 10%. However, over 24 h, time frames with sufficiently low artifact rates can be identified frequently, so that consultation can be based on these intervals. Time-domain parameters are checked for an approximate classification compared to a normal population to support the OHP’s assessment. Then, the color spectrogram is reviewed thoroughly together with the participant’s activity diary to identify resources and stressors which show distinct and different patterns in the spectrogram. Special interest is given to the existence of relaxation periods, i.e., if the night is a relaxation period (lower HR, higher HRV, distinct pattern in the HF band at night compared to day time) and if there are any other periods with markedly risen HRV, especially HF, during day time. The OHP concludes with some hypotheses of issues which the participant should improve, but importantly, the main conclusions can be drawn only at the end of the consultation when the OHP can merge these issues with the participant’s thoughts, feelings, and motivation.

The focus of the consultation is the linkage of HRV patterns to the diary to demonstrate both, strain and recovery periods in the HRV diagram and then to discuss these specific situations more deeply regarding the client’s emotions, thoughts, and bodily perceptions during these periods. This intervention aims at strengthening the perception of signs of the body, thoughts, and feelings, with a focus on relaxation periods. Other situations of the measurement day, especially periods of reduced HRV despite a normal or low HR, are also discussed similarly. The focus of the consultation is not to promote avoidance of these situations, but recovery after these situations. Regarding the already discussed resources, possible actions that could be taken next time for better recovery are discussed at the end. The consultation closes with overall feedback about the level of variability and a summary of conclusions resulting from the measurement and consultation, preferably developed together with the participant and not given as unidirectional advice. Details and examples of the consultation are given in (Jarczok et al., 2019).

### Data Sample

ECG data was not available for technical reasons for seven participants. A total of 17 ECGs showed more than 10% artifacts/non-sinus rhythm and were therefore excluded from HRV analyses. All other ECGs showed at least 90% sinus rhythm. Seven participants had a recording time shorter than 79,200 s and were therefore excluded from 24 h-analyses. Thus, the number of ECGs included in the analyses was 137.

One participant did not return the first questionnaire and 45 did not return the second questionnaire after the consultation, resulting in *N* = 167 for the first and *N* = 123 for the second questionnaire. As some participants did not submit a questionnaire for time point 1 but for time point 2, the complete analysis sample for hypothesis testing was *N* = 118.

Regarding the OHPs, *N* = 16 questionnaires are available for the first questionnaire and *N* = 9 for the final questionnaire, due to the ongoing training at study site C and D.

### Statistical Methods

The primary outcome is analyzed using Wilcoxon signed ranks tests. Effect size is calculated using Cohen’s d. All data management and statistics were conducted using SPSS Statistics for Windows, version 25 (SPSS Inc., Chicago, IL, United States). A *p*-value smaller than 0.05 (one-sided) was considered statistically significant. Due to the exploratory nature, no adjustments were made for cumulative alpha error due to multiple testing.

## Results

Between July 2018 and June 2019, *N* = 99 check-up consultations for managers were performed by the OHPs at study site A. All of them were offered the HRV-based consultation and *N* = 49 (49%) agreed to participate (see flow-chart Figure 1). Participants were not significantly different from non-participants regarding age and sex (all *p* > 0.05). At study site B, HRV-based consultation was offered not only to managers, but also to employees by written information available at the company’s medical service and not associated to a certain check-up. A total of 89 (74%) of the *N* = 118 measurements of study site B were performed because the employee/manager requested it and 24 (20%) because the OHP offered the measurement. Reasons for a direct offer by the OHP were reported tiredness, tension, sleep problems, stress levels reported as high and burdening, and somatic symptoms like dizziness, pain.

**FIGURE 2:**
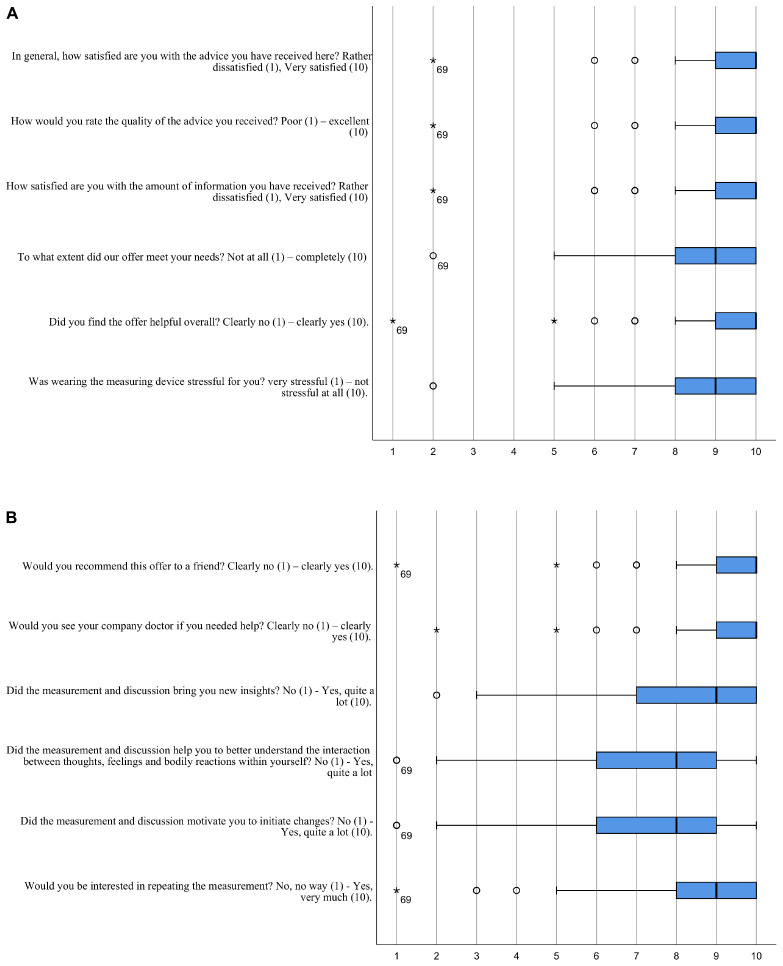
**(A,B)** Satisfaction of the participants with the consultation. Boxplot from *N* = 123 participants.

The combined sample of 168 participants was between 23 and 63 years old (mean = 45.5, SD = 9.8) and 68% were male (Table 1). A third of them indicated that they work 50 h or more per week. Almost half reported engaging in regular physical activities more than 2 h/week and every second participant rated his/her sleep as satisfying. Two-thirds reported knowledge about relaxation techniques, while about the same amount reported never practicing any relaxation techniques. Elevated depression scores were present in 21 (13%) and elevated anxiety scores in 21 (13%) participants. The average workability index was high (mean = 7.3, SD = 1.7, scale range from 0 to 10). The participants were highly satisfied with the consultation (median: 10; interquartile range: 9-10, scale range from 1 to 10) (see Figure 2). The SDNN-i of each 24 h series was classified using percentiles from a large (*N* > 7000) working cohort (see appendix for the percentiles in Supplementary Table 2). Eleven participants (8%) had values lower than the 10^*th*^ percentile and 40 participants (29%) had values lower than the 25th percentile. Associations between HRV percentiles and psychometric/sociodemographic data are shown in Supplementary Table 3.

**TABLE 1:** Descriptive.

	*N*	%	
CV risk factor count:	163		
0	131	80	
1	26	16	
2	5	3	
3	1	1	
Diagnosis count:	163		
0	104	64	
1	30	18	
2	16	10	
3 and more	13	8	
Women	57	35	
Current Smokers	14	8	
Weekly working hours: >50	52	31	
More than 2 h sport/week	69	41	
Relaxation method known	114	68	
Never using a relaxation method	103	62	
High quality sleep (subjective)	88	54	

	***N***	**Mean**	**SD**

Age (years)	167	45.5	9.8
BMI (kg/m^2^)	166	24.9	3.7
Workability	165	7.3	1.7
Cognitive irritation	166	11.3	4.8
Emotional irritation	166	14.2	5.9
PSS sum score	167	5.1	2.8
PHQ sum score	167	2.6	2.3

	***N***	**Median**	**Interquartile range**

Overall satisfaction with consultation	101	10	9–10
Recommendation to a friend	101	10	9–10
New insights	101	9	7–10
Improved psychosomatic perspective	101	8	6–9

**24 h HRV parameters**	***N***	**Median**	**Interquartile range**

HR (beats per minute)	137	74.81	69.78–79.87
SDNN (msec)	137	135.26	115.32–171.27
SDNN-i (msec)	137	60.12	50.64–73.64
SDANN (msec)	137	118.75	98.03–150.66
rMSSD (msec)	137	29.10	23.12–39.11
LF (msec^2^)	137	1,106.56	763.46–1,727.52
HF (msec^2^)	137	294.08	157.38–536.79
Total power (msec^2^)	137	3,594.64	2,641.10–5,238.65

**FIGURE 3:**
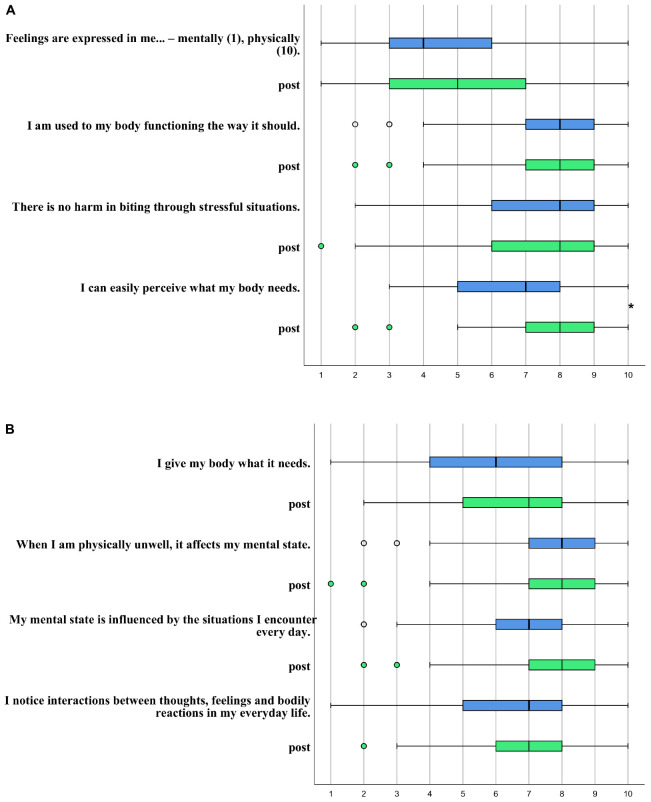
**(A,B)** Psychosomatic perceptions before (blue) and after (green, marked as “post”) the consultation. Boxplot from *N* = 118 participants. *p < 0.05. If not explicitly stated, the scale is rated between: Do not agree at all (1) – strongly agree (10).

Participants reported a significantly improved perception of their bodily needs after the consultation (from Median = 7, interquartile range 5–8 to Median = 8, interquartile range 7–9; scale range from 1 to 10; p < 0.001, Wilcoxon rank test; Cohen’s D = 0.49; *N* = 118) (see Figure 3).

A total of 16 OHPs were trained by our team. After the first training day, they felt sufficiently trained to conduct the HRV measurement (Median 5, interquartile range 4.25–5, scale between 1 = not at all and 6 = almost completely). During the regular meetings in the following months, the OHPs reported good acceptance of the HRV measurement. The effort to offer and explain the HRV measurement to potential participants was estimated as moderate (Median 5, interquartile range 3–8, scale between 1 = very large and 10 = very small). The procedure to equip participants, to return the HRV monitor, and transfer the recording to the local database was reported as time-consuming (about 30 min per participant). The procedure improved for the OHPs after they had instructed their medical staff to manage this. The reasons difficulties were encountered with the equipment was reported to be due to the exceptionally hot summer, and electrodes loosened quicker than normal and therefore started to move. This was improved by replacement with a textile chest belt with dry electrodes. Second, the electrodes were partially visible under light summer clothing that the participants found unpleasant. Logistic problems occurred on Fridays, as the participants did not want to conduct the measurement during their free weekend. Also, when participants worked at other sub-locations of study site A, they were unable to return the Holter monitor the next day.

The processing of ECG-data with the HRV software still took too much time for the OHPs (about 10 min per measurement; only technical processing, without interpretation) and we found no possibility to improve the process apart from changing the software to Cardiscope^TM^, which we did at the study sites B–D. Still, the question of the effort to analyze and interpret the HRV was answered as high (Median 3, interquartile range 1.5 to 4, scale between 1 = very large and 10 = very small). Discussing this issue further, OHPs reported that it needed about 10 cases to get into a routine and be more time-efficient, both with handling the software and interpreting the results. In the beginning, questions regarding interpretation of the results and communicating the results were frequent. Those with lesser cases still tended to feel insecure in analyzing, interpreting, and discussing the HRV graphs at the end of the year.

The extra time consumed by the HRV based consultation was reported as moderate (Median 4, interquartile range 2.5 to 4, scale between 1 = very high and 10 = very small). OHPs at study site A estimated an extra 15 min for HRV-based consultation adding this time to the pre-existing check-up consultation. The whole check-up examination and consultation took on average 2 1/2 h. At the other study sites, consultation times ranged between 30 min and one hour.

The OHPs reported the HRV measurement was, in general, helpful for consulting the participants (Median 4, interquartile range 2.5 to 4, scale between 1 = very helpful and 10 = not helpful at all), while they marked interesting cases where it was especially helpful, e.g., in the case of a young, hard-working female manager with a chronically ill child and tinnitus, which was rapidly assigned by her to her high strain. She felt quite bad about that because there was nothing she could change about her child and she did not want to reduce working hours as her job gave her high satisfaction. The results of the measurement were surprisingly positive, so she was encouraged to seek further medical help. A protracted otitis media and a perforated eardrum were detected and treated. Her tinnitus and her wellbeing improved. Another OHP reported that he was able to motivate a smoker with beginning arteriosclerosis and noticeably low HRV patterns at night to participate in a 1-week health training that is offered by the company.

Most OHPs wished to continue to use HRV-based consultation (Median 8; scale between 1 = not at all and 10 = yes, absolutely), but not for all check-ups, but selected cases like clients with sleeping problems, stress, or general fitness evaluation (free-text comments).

The OHPs reported that through the training, they felt empowered to continue applying HRV independently (Median 5, interquartile range 4.5 to 10, scale between 1 = not at all and 10 = yes, absolutely). The meetings with case studies were very helpful (median 10, interquartile range 9 to 10; scale between 1 = not at all and 10 = yes, absolutely) and the training met their expectations (median 8.5, interquartile range 8 to 10; scale between 1 = not at all and 10 = yes, absolutely). Most would recommend it to a colleague (median 8; interquartile range 6.25 to 10, scale between 1 = not at all and 10 = yes, absolutely). Still, insecurities in interpreting the results were reported mainly by those OHPs with less than 10 cases as reported above. Other OHPs stated that they found it difficult to conduct the consultation with the psychosomatic, resource-oriented focus. They requested more training in these special communication skills. Most of the OHPs reported that this kind of consultation cannot be shortened to less than 30 min, a time frame that is sometimes hard to offer if the workload is high.

Overall, the OHPs reported their impression that the participants were very satisfied with the consultations. Also, OHPs and participants (free-text comments) independently pointed out that the HRV measurement should be continued and offered to all employees; not necessarily during the check-up, but preferably for selected cases.

## Discussion

This first data from our interventional trial shows that measuring 24 h-HRV and consulting based on the results can improve the perception of the bodily needs of employees and managers. An appropriate perception of needs is a basis for healthy behavior. Participants were highly satisfied with the consultation and OHPs reported that the measurement of HRV is very well suited to enter into dialog with the managers and employees and to show interactions between situations, thoughts, feelings, and bodily reactions. Therefore, we conclude that a 24 h-HRV-measurement can be a feasible and effective approach for holistic, psychosomatic primary prevention in occupational medicine.

We want to mark that our intervention is based on increasing awareness of bodily signals and psychosomatic interactions, while also enhancing resources and confirming situations in which managers and employees successfully recovered from stressful situations. Especially, we wanted to avoid marking “stressful situations” as “dangerous”. We have achieved this goal, as stressful situations were not regarded as more harmful after compared with before the intervention (Figure 3, question 3). Most often, research results are classified under the headline “stress is bad for you”. Studies have reported links between both work stress, especially deadline stress, and also high-level acute episodes of anger or anxiety with triggering of acute MI (McCormack et al., 2016; Buckley et al., 2019). However, that’s a global finding and on an individual level, a comparable level of stress may be associated with heart disease in one person while another person may not be burdened at all. Beyond genetic predisposition, this probably depends on the frequency of exposure, intensity, and duration of the stressor. In other disciplines like cardiology, it turned out that the previously given common recommendation to do less physical exercise after being diagnosed with heart disease is counterproductive (Moraes-Silva et al., 2017). The circulatory system has to be used to function properly and to keep its functions – in other words, low to moderate, well-dosed stress appears essential for the system (when followed by recovery), while vigorous exertion is associated with increased CVD (Buckley et al., 2019). On a molecular level, recovery time played a pivotal role in a mouse model after traumatic stress exposure that triggered an acute heart injury. Interestingly, the repair process in the heart tissue was completed after 10 days. More importantly, group differences (long vs short exposure and long vs short recovery) were apparent in the recovery time but not in the stress exposure time, highlighting the important role of recovery time (Cho et al., 2014). Having this in mind, the presented consultation focuses on sufficient recovery (i.e., quality and quantity), not on avoiding stressful situations, i.e., avoiding chronic stress, not acute stressors – considering that this is primary prevention, the participants are healthy and the stressors are mild to moderate, far from the type of stressors causing post-traumatic stress disorder. As we focus on recovery after stressful situations and many participants show sufficient recovery, they actually can see on the graph that the stressful situation did not do them any harm and that they can handle it. We think that this change of mindset even has a health-promoting effect, because the stress at work is then no longer considered harmful, but rather like a vaccination empowering the individual do deal better with stress. Meichenbaum transferred the model of vaccination to stress and developed a “stress inoculation training,” claiming that regular exposure to mild stressors promotes resilience and the feeling of self-effectiveness (Meichenbaum and Deffenbacher, 1988). The importance of mindset on health is shown in another study by Crum who informed hotel room attendants that their work is a healthy exercise. Only this change in mindset without any behavioral changes led to a decrease in weight, blood pressure, and body fat compared to a control group who did not receive this information (Crum and Langer, 2007). We consider the changes found in psychosomatic attitudes and perception of the participants in our study as an indicator that the intervention positively changes their mindset, and we propose that this fact in itself may improve their health in addition to any behavioral changes we may have induced. We will further explore these hypotheses in our next study containing a repeated measurement after 3 months.

### Beyond This Study: What Are the Potential Mechanisms/Physiological Considerations

How can these mechanisms be linked to the ANS? Two very likely mechanisms appear to be pivotal. First, on the level of the brain, central autonomic processes, and processing shape the efferent projection activity via the lower brain regions such as to brain stem and further via the vagus nerve – that is the moment-to-moment evaluation of environmental threat and safety signals, its integration with memory, social functioning and emotion regulation, and also its projected trajectory into the near and distant future (Lane et al., 2009a, b; Thayer and Lane, 2009; Thayer et al., 2011; Holzman and Bridgett, 2017). If something is considered a threat or a safety signal is highly shaped by former experience, existing coping mechanisms, and actual beliefs. That’s how the mindset of a person (that is his or her beliefs) can shape bodily response in a moment-to-moment fashion. The diseases that should be modified by this pathway should be those of the organs addressed by efferent ANS activity. The efferent vagal activity can inhibit (indirectly) cytokine release from immune cells, also known as the cholinergic anti-inflammatory pathway, acting through the α7 nicotinic acetylcholine receptor (α7 nAChR) on macrophages (Huston and Tracey, 2011). As excessive pro-inflammatory cytokine release is common in many chronic and non-communicable disease conditions (including MI, CVD, rheumatic diseases, and depression), the latter pathway appears to be highly relevant in disease etiology (Huston and Tracey, 2011). This is a possible mechanism of how changes in mindset can change bodily reaction patterns like blood pressure via the ANS.

### Limitations

We do not know if the study sample is representative of the managers and employees working in these companies. It may be that our sample consists of managers and employees that care more for their health. We do not have data from the companies regarding the sex and age of all their managers and employees. However, we can compare our study population with other data from Germany. The present sample showed a healthier lifestyle and fewer diagnoses than men of their age in Germany which we expected for a sample with high socioeconomic status (Stringhini et al., 2017): Mean BMI was 25 kg/m^2^ and therefore lower than the mean BMI of 27 kg/m^2^ reported for German men between 40 and 60 years old (Mikrozensus, 2017). The sample contained very few smokers: 2% compared to 34% of men smoking in the German general population and about 23% in a German sample of middle-aged men with higher education (Lampert, 2011). Regarding psychometric variables, the PSS scale score was lower than in other samples of healthy men (Lavoie and Douglas, 2012; Vallejo et al., 2018) and the PHQ sum scores were comparable (Löwe et al., 2010). It is interesting that though our study sample had a relatively healthy lifestyle and low burden of disease, the percentage of managers with low HRV values was not lower than in the sample from a large (*N* > 7000) working cohort (see Supplementary Table 2). A noticeable characteristic of this sample was the long working hours reported. As long working hours correlate with reduced HRV (Park et al., 2001), this might partially explain this unexpected finding.

Regarding the training of the OHPs, it is a limitation that the evaluation is based on subjective assessment only. A more sophisticated approach would contain systematic qualitative analyses as well as an objective examination of the skills. This was beyond the scope of the funding of the present project.

Further, the supposed primary prevention effects of the approach presented here need to be confirmed by a long-term follow-up study to evaluate the potential health benefits for the participants.

### Training of OHPs

Regarding the OHPs, the training to interpret the 24 h-spectrogram of HRV and learn the necessary techniques for this consultation, as well as the consultations themselves, take time. Future workshops will contain two instead of one training day at the start. Our concept of repeated meetings turned out to be helpful to improve the skills and knowledge that the company doctors still needed. As they required more training in resource-oriented and psychosomatically focused consultations, we added this to the meetings. We found it very important to focus on this. Otherwise, the HRV measurement is easily used like other risk scores in the sense that the risk and advice are told to the client, but the client has gained no insights into his body and the interactions between body, mind, and environment.

It turned out clearly that the preparation of the measurement and the technical details of analyzing should be as least time-consuming as possible for the OHPs so that they could concentrate on what is important: the consultation of the client. Thus, involving the medical staff from the outset is very important.

Taken together, we show that a 24 h-HRV-measurement can be a feasible and effective approach for holistic, psychosomatic primary prevention in occupational medicine.

## Data Availability Statement

Due to data protection agreements, the research data cannot be uploaded to a public repository. However, the data can be reviewed upon request onsite at the Clinic for Psychosomatic Medicine and Psychotherapy (Ulm, Germany). Requests to access the datasets should be directed to EB, elisabeth.balint@uni-ulm.de.

## Ethics Statement

The studies involving human participants were reviewed and approved by Ethics Committee of Ulm University, Germany (ID 188/18). The participants provided their written informed consent to participate in this study.

## Author Contributions

MJ and EB: concept and design, acquisition, analysis, and interpretation of data, drafting of the manuscript, and statistical analysis. TB, HG, IB, DM, and JT: critical revision of the manuscript for important intellectual content. All authors read and approved the final manuscript.

## Conflict of Interest

IB received a fee for a HRV presentation (Training for doctors 09/2019 Dresden) from Firma Grünethal GmbH (Germany). The remaining authors declare that the research was conducted in the absence of any commercial or financial relationships that could be construed as a potential conflict of interest.

## References

[B1] Aeschbacher S. Schoen T. Dörig L. Kreuzmann R. Neuhauser C. Schmidt-Trucksäss A. (2017). Heart rate, heart rate variability and inflammatory biomarkers among young and healthy adults. *Ann. Med.* 49 32–41. 10.1080/07853890.2016.1226512 27534940

[B2] Airaksinen J. Jokela M. Virtanen M. Oksanen T. Koskenvuo M. Pentti J. (2018). Prediction of long-term absence due to sickness in employees: development and validation of a multifactorial risk score in two cohort studies. *Scand. J. Work. Environ. Health* 44 274–282. 10.5271/sjweh.3713 29363714

[B3] Backé E.-M. Seidler A. Latza U. Rossnagel K. Schumann B. (2012). The role of psychosocial stress at work for the development of cardiovascular diseases: a systematic review. *Int. Arch. Occup. Environ. Health* 85 67–79. 10.1007/s00420-011-0643-6 21584721 PMC3249533

[B4] Baevskiĭ R. M. (2002). [Analysis of variability of cardiac rhythm in space medicine]. *Fiziol. Cheloveka* 28 70–82. 11966235

[B5] Benarroch E. E. (2014). “Central autonomic network,” in *Autonomic Neurology*, ed. Benarroch E. (Oxford: Oxford University Press), 3–14. 10.1093/med/9780199920198.003.0001

[B6] Bernard C. (1867). *Lecture on the Physiology of the Heart and its Connections With the Brain, Delivered at the Sorbonne*, trans. Morel J. S. 1 Edn Savannah, GA: Purse & Son.

[B7] Bläsing D. (2017). Erfassung von individuellem beanspruchungserleben am arbeitsplatz über herzratenvariabilität im pflegebereichheart rate variability as an individual parameter to describe and explain stress experience of nursing staff. *Z. Arbeitswiss* 71 269–278. 10.1007/s41449-017-0082-7

[B8] Buccelletti E. Gilardi E. Scaini E. Galiuto L. Persiani R. Biondi A. (2009). Heart rate variability and myocardial infarction: systematic literature review and metanalysis. *Eur. Rev. Med. Pharmacol. Sci.* 13 299–307. 19694345

[B9] Buckley T. Soo Hoo S. Y. Shaw E. Hansen P. S. Fethney J. Tofler G. H. (2019). Triggering of acute coronary occlusion by episodes of vigorous physical exertion. *Hear. Lung Circ.* 28 1773–1779. 10.1016/j.hlc.2018.11.001 30555009

[B10] Chattipakorn N. Incharoen T. Kanlop N. Chattipakorn S. (2007). Heart rate variability in myocardial infarction and heart failure. *Int. J. Cardiol.* 120 289–296. 10.1016/j.ijcard.2006.11.221 17349699

[B11] Cho J. H. Lee I. Hammamieh R. Wang K. Baxter D. Scherler K. (2014). Molecular evidence of stress-induced acute heart injury in a mouse model simulating posttraumatic stress disorder. *Proc. Natl. Acad. Sci. U.S.A.* 111 3188–3193. 10.1073/pnas.1400113111 24516145 PMC3939897

[B12] Cohen S. Kamarck T. Mermelstein R. (1983). A global measure of perceived stress. *J. Heal. Soc Behav.* 24 385–396. 10.2307/2136404 6668417

[B13] Crum A. J. Langer E. J. (2007). Mind-set matters: exercise and the placebo effect. *Psychol. Sci.* 18 165–171. 10.1111/j.1467-9280.2007.01867.x 17425538

[B14] DeStatis (Hrsg.) (2017). Cardiovascular Diseases Cause Highest Costs. *Press Release No. 347 29*. September. 2017.

[B15] Elovainio M. Kivimäki M. Puttonen S. Lindholm H. Pohjonen T. Sinervo T. (2006). Organisational injustice and impaired cardiovascular regulation among female employees. *Occup. Environ. Med.* 63 141–144. 10.1136/oem.2005.019737 16421394 PMC2078070

[B16] Eurostat (Hrsg.) (2020). *Employment – Annual Statistics. Employ. (as % Population Aged 20 to 64).* Luxembourg: Eurostat.

[B17] Folkman S. Lazarus R. S. Gruen R. J. DeLongis A. (1986). Appraisal, coping, health status, and psychological symptoms. *J. Pers. Soc. Psychol.* 50 571–579. 10.1037/0022-3514.50.3.571 3701593

[B18] Herr R. M. Li J. Bosch J. a. Schmidt B. Dejoy D. M. Fischer J. E. (2012). Psychometric properties of a German organizational justice questionnaire (G-OJQ) and its association with self-rated health: findings from the Mannheim Industrial Cohort Studies (MICS). *Int. Arch. Occup. Environ. Health* 87 85–93. 10.1007/s00420-012-0839-4 23266905

[B19] Holzman J. B. Bridgett D. J. (2017). Heart rate variability indices as bio-markers of top-down self-regulatory mechanisms: a meta-analytic review. *Neurosci. Biobehav. Rev.* 74 233–255. 10.1016/j.neubiorev.2016.12.032 28057463

[B20] Huikuri H. V. Stein P. K. (2013). Heart rate variability in risk stratification of cardiac patients. *Prog. Cardiovasc. Dis.* 56 153–159. 10.1016/j.pcad.2013.07.003 24215747

[B21] Huston J. M. Tracey K. J. (2011). The pulse of inflammation: heart rate variability, the cholinergic anti-inflammatory pathway and implications for therapy. *J. Intern. Med.* 269 45–53. 10.1111/j.1365-2796.2010.02321.x 21158977 PMC4527046

[B22] Jarczok M. N. Guendel H. McGrath J. Balint E. M. (2019). “Circadian rhythms of the autonomic nervous system – scientific implication and practical implementation,” in *Chronobiology*, ed. Svorc P. (London: IntechOpen).

[B23] Jarczok M. N. Jarczok M. Mauss D. Koenig J. Li J. Herr R. M. (2013a). Autonomic nervous system activity and workplace stressors-A systematic review. *Neurosci. Biobehav. Rev.* 37 1810–1823. 10.1016/j.neubiorev.2013.07.004 23891906

[B24] Jarczok M. N. Jarczok M. Thayer J. F. (2020). “Work stress and autonomic nervous system activity,” in *Handbook of Socioeconomic Determinants of Occupational Health – From Macro-level to Micro-Level Evidence*, ed. Theorell T. (Basel: Springer International Publishing), 690 10.1007/978-3-030-05031-3_27-1

[B25] Jarczok M. N. Kleber M. E. Koenig J. Loerbroks A. Herr R. M. Hoffmann K. (2015). Investigating the mechanisms of self-rated health: heart rate variability is more strongly associated than other frequently used biomarkers in a cross sectional occupational sample. *PLoS One* 10:e0117196. 10.1371/journal.pone.0117196 25693164 PMC4333766

[B26] Jarczok M. N. Koenig J. Mauss D. Fischer J. E. Thayer J. F. (2014). Lower heart rate variability predicts increased level of C-reactive protein 4 years later in healthy, nonsmoking adults. *J. Intern. Med.* 276 667–671. 10.1111/joim.12295 25141771

[B27] Jarczok M. N. Li J. Mauss D. Fischer J. E. Thayer J. F. (2013b). Heart rate variability is associated with glycemic status after controlling for components of the metabolic syndrome. *Int. J. Cardiol.* 167 855–861. 10.1016/j.ijcard.2012.02.002 22386703

[B28] Järvelin-Pasanen S. Sinikallio S. Tarvainen M. P. (2019). Heart rate variability and occupational stress— systematic review. *Ind. Health* 56 500–511. 10.2486/indhealth.2017-0190 29910218 PMC6258751

[B29] Kaikkonen P. Hynynen E. Mann T. Rusko H. Nummela A. (2010). Can HRV be used to evaluate training load in constant load exercises? *Eur. J. Appl. Physiol.* 108 435–442. 10.1007/s00421-009-1240-1 19826833

[B30] Karasek R. (1979). Job demands, job decision latitude and mental strain?: implications for job redesign. *Adm. Sci. Q.* 24 285–308. 10.2307/2392498 30315367

[B31] Kemp A. H. Brunoni A. R. Santos I. S. Nunes M. A. Dantas E. M. Carvalho (2014). Effects of depression, anxiety, comorbidity, and antidepressants on resting-state heart rate and its variability: an ELSA-Brasil cohort baseline study. *Am. J. Psychiatry* 171 1328–1334. 10.1176/appi.ajp.2014.13121605 25158141

[B32] Klein E. M. Brähler E. Dreier M. Reinecke L. Müller K. W. Schmutzer G. (2016). The german version of the perceived stress scale – psychometric characteristics in a representative German community sample. *BMC Psychiatry* 16:1–10. 10.1186/s12888-016-0875-9 27216151 PMC4877813

[B33] Kristal-Boneh E. Raifel M. Froom P. Ribak J. (1995). Heart rate variability in health and disease. *Scand. J. Work. Environ. Health* 21 85–95. 10.5271/sjweh.15 7618063

[B34] Kroenke K. Spitzer R. L. Williams J. B. W. Löwe B. (2009). An ultra-brief screening scale for anxiety and depression: the PHQ-4. *Psychosomatics* 50 613–621. 10.1176/appi.psy.50.6.613 19996233

[B35] Lakusic N. Mahovic D. Sonicki Z. Slivnjak V. Baborski F. (2013). Outcome of patients with normal and decreased heart rate variability after coronary artery bypass grafting surgery. *Int. J. Cardiol.* 166 516–518. 10.1016/j.ijcard.2012.04.040 22560918

[B36] Lampert T. (2011). Rauchen – aktuelle entwicklungen bei erwachsenen. *GBE Kompakt Zahl Trends Gesundheitsbericht. Bundes* 2 1–9.

[B37] Lane R. D. McRae K. Reiman E. M. Chen K. Ahern G. L. Thayer J. F. (2009a). Neural correlates of heart rate variability during emotion. *Neuroimage* 44 213–222. 10.1016/j.neuroimage.2008.07.056 18778779

[B38] Lane R. D. Waldstein S. R. Critchley H. D. Derbyshire S. W. G. Drossman D. A. Wager T. D. (2009b). The rebirth of neuroscience in psychosomatic medicine, Part II: clinical applications and implications for research. *Psychosom. Med.* 71 135–151. 10.1097/PSY.0b013e318198a11f 19196806

[B39] Lavoie J. A. A. Douglas K. S. (2012). The perceived stress scale: evaluating configural, metric and scalar invariance across mental health status and gender. *J. Psychopathol. Behav. Assess.* 34 48–57. 10.1007/s10862-011-9266-1

[B40] Lindström J. Tuomilehto J. (2003). The diabetes risk score: a practical tool to predict type 2 diabetes risk. *Diabetes Care* 26 725–731. 10.2337/diacare.26.3.725 12610029

[B41] Löwe B. Kroenke K. Gräfe K. (2005). Detecting and monitoring depression with a two-item questionnaire (PHQ-2). *J. Psychosom. Res.* 58 163–171. 10.1016/j.jpsychores.2004.09.006 15820844

[B42] Löwe B. Wahl I. Rose M. Spitzer C. Glaesmer H. Wingenfeld K. (2010). A 4-item measure of depression and anxiety: Validation and standardization of the patient health questionnaire-4 (PHQ-4) in the general population. *J. Affect. Disord.* 122 86–95. 10.1016/j.jad.2009.06.019 19616305

[B43] Lundberg U. (2015). Work conditions and back pain problems. *Stress Heal.* 31 1–4. 10.1002/smi.2633 25641822

[B44] Maslach C. Jackson S. E. (1981). The measurement of experienced burnout. *J. Organ. Behav.* 2 99–113. 10.1002/job.4030020205

[B45] May A. Buckley T. (2019). *Matchfit: The Complete Manual to get Your Body and Brain fit For Work and Fit for Life*, 1 Edn Sydney, NSW: Brio Books.

[B46] McCormack C. Tofler G. Soo Hoo S. Hansen P. Buckley T. (2016). Onset of acute coronary syndrome following work-related events. *Heart. Lung Circ.* 25:S323 10.1016/j.hlc.2016.06.012

[B47] Meichenbaum D. H. Deffenbacher J. L. (1988). Stress inoculation training. *Couns. Psychol.* 16 69–90. 10.1177/0011000088161005

[B48] Mercedes R. C. Duanping L. Evans G. W. Cascio W. E. Chambless L. E. Rosamond W. D. (2002). Does the cardiac autonomic response to postural change predict incident coronary heart disease and mortality? The atherosclerosis risk in communities study – pubmed. *Am. J. Epidemiol.* 155 48–56. 10.1093/aje/155.1.48 11772784

[B49] Mikrozensus (2017). *Gesundheitszustand und -Relevantes Verhalten: Körpermaße nach Altersgruppen: Männer.* Available online at: https://www.destatis.de/DE/Themen/Gesellschaft-Umwelt/Gesundheit/Gesundheitszustand-Relevantes-Verhalten/Tabellen/koerpermasse-maenner.html (accessed May 29, 2020).

[B50] Mohr G. Rigotti T. Müller A. (2005). Irritation – Ein instrument zur erfassung psychischer beanspruchung im arbeitskontext. Skalen- und itemparameter aus 15 studien. *Zeitschr. Arb. Organ* 49 44–48. 10.1026/0932-4089.49.1.44

[B51] Moraes-Silva I. C. Rodrigues B. Coelho-Junior H. J. Feriani D. J. Irigoyen M.-C. (2017). “Myocardial infarction and exercise training: evidence from basic science,” in *Advances in Experimental Medicine and Biology*, ed. Xiao J. (New York, NY: Springer), 139–153. 10.1007/978-981-10-4307-9_9 29022262

[B52] Odegaard A. O. Koh W. P. Gross M. D. Yuan J. M. Pereira M. A. (2011). Combined lifestyle factors and cardiovascular disease mortality in chinese men and women: the singapore chinese health study. *Circulation* 124 2847–2854. 10.1161/CIRCULATIONAHA.111.048843 22104554 PMC3400937

[B53] OECD (Hrsg.) (2020). *Health Expenditures and Financing. Jt. OECD, EUROSTAT WHO Heal. Accounts SHA Quest.* Paris: OCED.

[B54] Park J. Kim Y. Cho Y. Woo K.-H. Chung H. K. Iwasaki K. (2001). Regular overtime and cardiovascular functions. *Ind. Health* 39 244–249. 10.2486/indhealth.39.244 11500000

[B55] Robert Koch-Institut (Hrsg.) (2014). *Chronisches Kranksein. Faktenblatt zu GEDA 2012: Ergebnisse der Studie? Gesundheit in Deutschland Aktuell 2012.* Berlin: RKI.

[B56] Rückholdt M. Tofler G. H. Randall S. Buckley T. (2019). Coping by family members of critically ill hospitalised patients: an integrative review. *Int. J. Nurs. Stud.* 97 40–54. 10.1016/j.ijnurstu.2019.04.016 31132688

[B57] Sammito S. Thielmann B. Seibt R. Klussmann A. Weippert M. Böckelmann I. (2015). Guideline for the application of heart rate and heart rate variability in occupational medicine and occupational science. *ASU Int.* 2015 1–29. 10.17147/asui.2015-06-09-03 PMC11089808 38741189

[B58] Sandercock G. R. H. Brodie D. A. (2006). The role of heart rate variability in prognosis for different modes of death in chronic heart failure. *Pacing Clin. Electrophysiol.* 29 892–904. 10.1111/j.1540-8159.2006.00457.x 16923007

[B59] Schneiderman N. Ironson G. Siegel S. D. (2005). Stress and health: psychological, behavioral, and biological determinants. *Annu. Rev. Clin. Psychol.* 1 607–628. 10.1146/annurev.clinpsy.1.102803.144141 17716101 PMC2568977

[B60] Schuster A. K. Fischer J. E. Thayer J. F. Mauss D. Jarczok M. N. (2016). Decreased heart rate variability correlates to increased cardiovascular risk. *Int. J. Cardiol.* 203 728–730. 10.1016/j.ijcard.2015.11.027 26587729

[B61] Schwerdtfeger B. Reif R. Günthner W. A. Klinker G. Hamacher D. Schega L. (2009). “Pick-by-vision: a first stress test,” in *Proceedings of the 8th IEEE International Symposium on Mixed and Augmented Reality, ISMAR 2009*, Orlando, FL, 115–124. 10.1109/ISMAR.2009.5336484

[B62] Seeman T. E. Singer B. H. Rowe J. W. Horwitz R. I. McEwen B. S. (1997). Price of adaptation–allostatic load and its health consequences. MacArthur studies of successful aging. *Arch. Intern. Med.* 157 2259–2268. 10.1001/archinte.157.19.2259 9343003

[B63] Shaffer F. Ginsberg J. P. (2017). An Overview of heart rate variability metrics and norms. *Front. Public Heal.* 5:258. 10.3389/fpubh.2017.00258 29034226 PMC5624990

[B64] Siegrist J. (1996). *Soziale Krisen und Gesundheit?: Eine Theorie der Gesundheitsförderung am Beispiel von Herz-Kreislauf-Risiken im Erwerbsleben.* Göttingen: Hogrefe Verl. für Psychologie.

[B65] Stalder T. Kirschbaum C. Kudielka B. M. Adam E. K. Pruessner J. C. Wüst S. (2016). Psychoneuroendocrinology assessment of the cortisol awakening response: expert consensus guidelines. *Psychoneuroendocrinology* 63 414–432. 10.1016/j.psyneuen.2015.10.010 26563991

[B66] Stringhini S. Carmeli C. Jokela M. Avendaño M. Muennig P. Guida F. (2017). Socioeconomic status and the 25×25 risk factors as determinants of premature mortality: a multicohort study and meta-analysis of 1°7 million men and women. *Lancet* 389 1229–1237. 10.1016/S0140-6736(16)32380-7 28159391 PMC5368415

[B67] Thayer J. F. Åhs F. Fredrikson M. Sollers Iii J. J. Wager T. D. (2011). A meta-analysis of heart rate variability and neuroimaging studies: implications for heart rate variability as a marker of stress and health. *Neurosci. Biobehav. Rev.* 36 747–756. 10.1016/j.neubiorev.2011.11.009 22178086

[B68] Thayer J. F. Lane R. D. (2000). A model of neurovisceral integration in emotion regulation and dysregulation. *J. Affect. Disord.* 61 201–216. 10.1016/s0165-0327(00)00338-4 11163422

[B69] Thayer J. F. Lane R. D. (2007). The role of vagal function in the risk for cardiovascular disease and mortality. *Biol. Psychol.* 74 224–242. 10.1016/j.biopsycho.2005.11.013 17182165

[B70] Thayer J. F. Lane R. D. (2009). Claude Bernard and the heart-brain connection: further elaboration of a model of neurovisceral integration. *Neurosci. Biobehav. Rev.* 33 81–88. 10.1016/j.neubiorev.2008.08.004 18771686

[B71] Theorell T. Hammarström A. Aronsson G. Träskman B. L. Grape T. Hogstedt C. (2015). A systematic review including meta-analysis of work environment and depressive symptoms. *BMC Public Health* 15:738. 10.1186/s12889-015-1954-4 26232123 PMC4522058

[B72] Togo F. Takahashi M. (2009). Heart rate variability in occupational health - a systematic review. *Ind. Health* 47 589–602. 10.2486/indhealth.47.589 19996534

[B73] Vallejo M. A. Vallejo-Slocker L. Fernández-Abascal E. G. Mañanes G. (2018). Determining factors for stress perception assessed with the Perceived Stress Scale (PSS-4) in Spanish and other European samples. *Front. Psychol.* 9:37. 10.3389/fpsyg.2018.00037 29434563 PMC5791241

[B74] Warttig S. L. Forshaw M. J. South J. White A. K. (2013). New, normative, English-sample data for the Short Form Perceived Stress Scale (PSS-4). *J. Health Psychol.* 18 1617–1628. 10.1177/1359105313508346 24155195

[B75] Wilson P. W. F. D’Agostino R. B. Levy D. Belanger A. M. Silbershatz H. Kannel W. B. (1998). Prediction of coronary heart disease using risk factor categories. *Circulation* 97 1837–1847. 10.1161/01.CIR.97.18.1837 9603539

[B76] World Health Organisation (2005). *Preventing Chronic Diseases?: A Vital Investment?: WHO Global Report.* Geneva: WHO.

[B77] Wulsin L. Herman J. Thayer J. F. (2018). Stress, autonomic imbalance, and the prediction of metabolic risk: a model and a proposal for research. *Neurosci. Biobehav. Rev.* 86 12–20. 10.1016/j.neubiorev.2017.12.010 29277456

