# Supplementary material for: 24 h-Heart Rate Variability as a Communication Tool for a Personalized Psychosomatic Consultation in Occupational Health
Source: Front Neurosci. 2021 Feb 11;15:600865. doi: 10.3389/fnins.2021.600865 (PMC7905098; doi:10.3389/fnins.2021.600865)
Supplement: Supplementary file 1 [file Table_1.pdf]

Supplementary Table 1: Overview of training for the OHPs

Content of Training day 1 (8 hours):

- Introduction into HRV: basic physiology, time domain and frequency domain parameters, color spectrogram
- Handling of ECG device, electrodes, and chest belt
- Handling of HRV software incl. practical exercises with ECG data
- Handling of artifacts incl. practical exercises with ECG data
- Impact of diseases, medication, sleep and stress on HRV
- Modulation of HRV by behavior change
- Interpretation of HRV incl. practical exercises with ECG data and additional client information
- Resource-oriented Consultation
- Feedback and evaluation of the day

Case review group (2 hours at month 3, 6, and 12)

- Warm-up – Questions and Answers
- Semi-Structured discussion with the following questions from the supervisors:
  - What were the hurdles, what helped in presenting the project or HRV measurement?
  - How is the reaction of managers/employees if the measurement is suggested?
  - What difficulties did you have in returning the devices and questionnaires? What helped?
  - What difficulties did you have in evaluating the HRV measurement?
  - What were the hurdles, what helped regarding the consultation? Particularly difficult cases? Really fun cases?
  - What else would you need in terms of information, knowledge, skills… to improve the consultation?
  - Do you currently feel that HRV measurement is helpful and enriching the check-up/consultations? Why yes/no?
  - Do you have the impression that it achieves its goal of making managers/employees aware of a psychosomatic view? Why yes/no?
  - Was the questionnaire provided useful for the consultation?
- Presentation of 2-3 cases by OHPs with group discussion and input from the supervisors

Jarczok MN, Buckley T, Guendel HO, Boeckelmann I, Mauss D, Thayer JF and Balint EM (2021) 24 h-Heart Rate Variability as a Communication Tool for a Personalized Psychosomatic Consultation in Occupational Health. Front. Neurosci. 15:600865. doi: 10.3389/fnins.2021.600865 2021-01
