# Supplementary material for: 24 h-Heart Rate Variability as a Communication Tool for a Personalized Psychosomatic Consultation in Occupational Health
Source: Front Neurosci. 2021 Feb 11;15:600865. doi: 10.3389/fnins.2021.600865 (PMC7905098; doi:10.3389/fnins.2021.600865)
Supplement: Supplementary file 2 [file Table_2.pdf]

Supplementary Table 2: RMSSD, SDNN-i and Heart Rate from 24h ECG recordings in voluntary, predominantly healthy employees (MICS-Study)

Percentiles from average 5-min **RMSSD** (msec) values from 24h ECG recordings in voluntary, predominantly healthy employees (MICS-Study)

**Males**		Percentile										
Age group	**N**	**p1**	**p5**	**p10**	**p25**	**p50**	**p75**	**p90**	**p95**	**p99**	**Mean**	**SD**
**18/24**	125	18.5	26.6	28.1	38.1	47.4	58.8	70.2	80.4	94.5	48.8	16.8
**25/29**	476	17.8	23.5	26.8	33.6	41.3	52.2	65.4	72.8	86.5	44.1	15.0
**30/34**	638	15.5	19.8	22.2	28.8	36.5	45.6	56.5	64.7	73.1	38.2	13.3
**35/39**	739	12.7	17.2	19.8	25.1	31.1	39.5	50.3	57.7	70.8	33.5	12.2
**40/44**	775	11.6	15.8	18.0	22.4	28.2	35.4	44.1	49.3	65.7	29.9	10.6
**45/49**	1086	9.4	13.8	15.8	19.7	25.0	31.4	38.3	42.8	56.6	26.3	9.4
**50/54**	958	8.9	11.9	14.3	17.6	21.6	27.0	33.7	38.1	49.1	23.0	8.2
**55/59**	699	7.7	11.0	12.6	15.9	20.1	24.7	31.3	36.0	45.9	21.1	7.6
**60/65**	235	9.2	10.5	12.4	15.6	19.1	24.4	29.8	35.5	43.8	20.5	7.5
**Total**	5731	9.7	13.5	15.8	20.2	26.8	36.1	47.2	55.7	71.3	29.6	13.2

**Females**												
Age group	**N**	**p1**	**p5**	**p10**	**p25**	**p50**	**p75**	**p90**	**p95**	**p99**	**Mean**	**SD**
**18/24**	97	13.9	20.1	24.1	28.5	36.7	46.6	59.5	73.0	95.7	39.6	16.1
**25/29**	231	18.9	21.0	24.0	28.7	36.6	50.1	59.6	65.6	88.7	39.9	14.7
**30/34**	235	12.7	18.0	19.7	26.3	34.4	44.3	52.0	57.6	71.1	35.8	12.9
**35/39**	133	12.7	18.3	20.9	24.7	29.4	37.8	50.9	57.0	66.4	32.6	11.7
**40/44**	203	11.2	15.2	17.2	21.5	26.8	34.4	42.9	48.6	60.2	28.7	10.6
**45/49**	218	8.9	12.1	15.9	19.8	23.9	30.0	39.0	44.2	55.7	25.7	9.3
**50/54**	149	9.3	12.4	14.1	17.4	21.2	26.7	33.2	38.1	43.9	22.8	7.9
**55/59**	122	10.6	11.7	13.2	15.6	19.4	26.2	33.0	36.8	49.0	21.9	8.4
**60/65**	27	9.2	10.5	13.3	15.4	20.8	25.2	33.5	41.3	43.5	21.6	8.4
**Total**	1415	10.1	14.2	16.6	21.5	28.5	38.2	49.8	57.1	72.6	31.0	13.4

Citation: *Jarczok MN. Buckley T. Guendel HO. Boeckelmann I. Mauss D. Thayer JF and Balint EM (2021) 24 h-Heart Rate Variability as a Communication Tool for a Personalized Psychosomatic Consultation in Occupational Health. Front. Neurosci. 15:600865. doi: 10.3389/fnins.2021.600865*

Supplementary Table 2: RMSSD, SDNN-i and Heart Rate from 24h ECG recordings in voluntary, predominantly healthy employees (MICS-Study)

Percentiles from average 5-min **SDNN-i** (msec) values from 24h ECG recordings in voluntary, predominantly healthy employees (MICS-Study)

**Males**		Percentile										
Age group	**N**	**p1**	**p5**	**p10**	**p25**	**p50**	**p75**	**p90**	**p95**	**p99**	**Mean**	**SD**
**18/24**	125	42.9	53.8	58.7	71.2	82.5	97.4	109.1	113.8	126.7	83.9	19.3
**25/29**	476	46.9	55.8	60.1	68.7	80.5	93.1	106.5	114.4	127.3	81.8	17.8
**30/34**	638	41.2	50.2	55.2	64.3	74.3	86.5	99.6	105.9	117.7	76.0	16.6
**35/39**	739	36.8	46.3	50.7	60.1	70.2	82.2	95.1	102.3	114.2	71.8	17.0
**40/44**	775	34.5	44.9	48.4	56.6	67.6	77.9	88.6	96.7	106.6	67.9	15.6
**45/49**	1086	31.9	40.0	44.0	52.4	62.3	71.8	84.0	89.4	105.3	63.0	15.2
**50/54**	958	28.5	37.4	41.1	48.0	57.1	66.7	75.5	82.8	97.5	58.0	13.9
**55/59**	699	24.5	33.9	37.3	45.4	53.8	62.3	72.2	78.6	92.2	54.5	13.4
**60/65**	235	29.5	33.4	37.4	44.0	52.3	60.5	70.1	74.1	97.0	52.8	13.0
**Total**	5731	31.7	39.6	44.6	53.3	64.4	76.8	89.8	99.2	113.6	66.0	17.9

**Females**												
Age group	**N**	**p1**	**p5**	**p10**	**p25**	**p50**	**p75**	**p90**	**p95**	**p99**	**Mean**	**SD**
**18/24**	97	39.7	45.4	49.0	55.4	63.8	73.2	88.4	95.8	117.6	66.6	15.5
**25/29**	231	43.0	45.7	49.3	56.3	68.1	79.6	90.0	93.6	112.3	69.0	15.7
**30/34**	235	34.5	42.4	46.0	53.9	65.2	76.6	84.7	90.9	100.4	65.6	15.4
**35/39**	133	40.9	43.6	46.4	54.7	62.7	72.1	83.2	91.9	104.8	64.1	14.6
**40/44**	203	36.2	41.5	44.9	50.9	57.7	68.5	79.9	85.9	98.9	60.4	13.8
**45/49**	218	29.2	36.4	39.6	48.3	56.1	65.2	73.8	84.9	88.8	57.0	13.8
**50/54**	149	25.3	32.7	35.7	44.0	52.1	62.0	71.0	80.3	87.9	53.4	13.7
**55/59**	122	28.3	30.1	33.6	40.8	49.1	59.8	66.2	71.1	85.2	50.3	12.8
**60/65**	27	28.2	28.4	32.7	41.7	52.4	58.8	68.3	70.1	77.0	51.2	12.3
**Total**	1415	29.0	37.6	42.6	50.0	59.8	70.8	81.9	88.9	101.4	61.1	15.7

Citation: *Jarczok MN. Buckley T. Guendel HO. Boeckelmann I. Mauss D. Thayer JF and Balint EM (2021) 24 h-Heart Rate Variability as a Communication Tool for a Personalized Psychosomatic Consultation in Occupational Health. Front. Neurosci. 15:600865. doi: 10.3389/fnins.2021.600865*

Supplementary Table 2: RMSSD, SDNN-i and Heart Rate from 24h ECG recordings in voluntary, predominantly healthy employees (MICS-Study)

Percentiles from average 5-min **Heart Rate (BPM)** values from 24h ECG recordings in voluntary, predominantly healthy employees (MICS-Study)

**Males**		Percentile										
Age group	**N**	**p1**	**p5**	**p10**	**p25**	**p50**	**p75**	**p90**	**p95**	**p99**	**Mean**	**SD**
**18/24**	125	56.7	59.9	63.4	67.6	72.8	79.7	84.7	89.5	92.9	73.5	8.6
**25/29**	476	55.6	60.6	62.7	67.4	72.6	77.0	82.9	85.4	92.0	72.6	7.7
**30/34**	638	57.8	61.6	64.2	67.9	72.6	77.8	83.1	86.9	93.6	73.2	7.7
**35/39**	739	55.3	59.6	62.0	67.3	72.8	78.7	84.5	88.7	95.3	73.2	8.6
**40/44**	775	55.0	60.4	62.6	67.7	73.6	78.5	84.1	86.9	93.0	73.4	8.1
**45/49**	1086	54.9	60.0	62.1	67.4	73.3	78.9	84.4	87.3	93.1	73.3	8.5
**50/54**	958	54.7	59.7	62.2	67.1	72.6	78.8	84.6	87.3	93.7	73.1	8.6
**55/59**	699	53.6	58.1	61.3	67.1	72.7	78.0	84.3	87.4	95.6	72.6	8.7
**60/65**	235	55.3	59.8	62.2	66.7	71.8	77.2	82.1	86.5	89.5	72.0	7.8
**Total**	5731	55.2	59.9	62.3	67.4	72.9	78.4	84.0	87.3	93.4	73.0	8.3

**Females**												
Age group	**N**	**p1**	**p5**	**p10**	**p25**	**p50**	**p75**	**p90**	**p95**	**p99**	**Mean**	**SD**
**18/24**	97	60.7	66.8	70.8	76.7	81.1	85.2	91.9	92.5	95.7	80.9	7.5
**25/29**	231	61.7	64.5	68.0	72.0	77.8	83.0	88.1	91.7	94.8	78.0	7.8
**30/34**	235	61.4	65.3	68.3	72.3	76.9	82.2	86.9	90.4	99.6	77.5	7.6
**35/39**	133	58.5	64.4	67.4	72.6	78.0	81.6	86.0	87.2	92.4	76.9	7.3
**40/44**	203	59.7	63.9	67.0	71.8	77.9	82.8	87.6	89.9	93.2	77.3	7.9
**45/49**	218	59.7	64.3	67.1	70.9	76.8	82.5	88.0	90.1	93.8	77.1	8.0
**50/54**	149	58.5	62.5	66.5	70.4	76.3	81.5	87.8	89.8	95.2	76.4	8.0
**55/59**	122	62.9	65.7	66.7	70.9	75.2	81.3	85.0	88.4	94.4	75.9	7.4
**60/65**	27	58.1	65.0	66.0	69.0	75.4	79.4	84.6	84.8	89.3	75.2	7.1
**Total**	1415	59.8	64.6	67.3	71.9	77.4	82.5	87.6	90.2	95.2	77.4	7.8

Citation: *Jarczok MN. Buckley T. Guendel HO. Boeckelmann I. Mauss D. Thayer JF and Balint EM (2021) 24 h-Heart Rate Variability as a Communication Tool for a Personalized Psychosomatic Consultation in Occupational Health. Front. Neurosci. 15:600865. doi: 10.3389/fnins.2021.600865*
