# Supplementary material for: 24 h-Heart Rate Variability as a Communication Tool for a Personalized Psychosomatic Consultation in Occupational Health
Source: Front Neurosci. 2021 Feb 11;15:600865. doi: 10.3389/fnins.2021.600865 (PMC7905098; doi:10.3389/fnins.2021.600865)
Supplement: Supplementary file 3 [file Table_3.pdf]

Supplementary Table 3: Associations between HRV percentiles and psychometric/sociodemographic data

	SDNN-i < 25th percentile		SDNN-i >= 25th percentile		
N (%)	40	29%	97	71%	
	N	%	N	%	p (Fisher's exact test, one-sided)
**CV risk factors min. 1**	12	31%	13	14%	**.023**
**diagnoses min. 1**	18	46%	27	29%	**.043**
female gender	13	33%	34	35%	n.s.
weekly working hours: >50	16	40%	26	27%	.095
**more than 2h sport/week**	7	18%	47	49%	**.001**
relaxation method known	30	75%	65	67%	n.s.
never using a relaxation method	26	65%	57	59%	n.s.
high quality sleep (subjective)	21	53%	53	56%	n.s.

Reading example: Of the group of managers with HRV values lower than the 25th percentile, 31% have at least one CV risk factor, while in the group of managers with higher HRV values, only 14% have a CV risk factor.

	SDNN-i <10th percentile		SDNN-i >= 10th percentile		
N (%)	11	8%	126	92%	
	N	%	N	%	p (Fisher's exact test, one-sided)
**CV risk factors min. 1**	6	55%	19	16%	**.006**
diagnoses min. 1	5	46%	40	33%	n.s.
female gender	2	18%	45	36%	n.s.
weekly working hours: >50	6	55%	36	29%	.077
more than 2h sport/week	2	18%	52	41%	n.s.
relaxation method known	7	64%	88	70%	n.s.
never using a relaxation method	7	64%	76	60%	n.s.
high quality sleep (subjective)	6	55%	68	55%	n.s.

Jarczok MN, Buckley T, Guendel HO, Boeckelmann I, Mauss D, Thayer JF and Balint EM (2021) 24 h-Heart Rate Variability as a Communication Tool for a Personalized Psychosomatic Consultation in Occupational Health. Front. Neurosci. 15:600865. doi: 10.3389/fnins.2021.600865
2021-01

	SDNN-i < 25th percentile			SDNN-i >= 25th percentile			Mann Whitney U test P (two-sided)
	N	mean	SD	N	mean	SD	
age [years]	40	43.2	10.0	97	45	9.8	n.s.
BMI [kg/m²]	40	25.8	4.5	96	24.6	3.3	n.s.
work ability index	40	7.6	1.5	96	7.1	2	n.s.
cognitive irritation	40	12.2	4.4	97	11.2	4.9	n.s.
emotional irritation	40	12.9	4.9	97	15	6.1	.089
PSS sum score	40	5.3	2.1	97	5.2	3.1	n.s.
PHQ sum score	40	2.4	2.0	97	2.8	2.3	n.s.

	SDNN-i <10th percentile			SDNN-i >= 10th percentile			Mann Whitney U test (two-sided)
	N	mean	SD	N	mean	SD	
age [years]	11	44	10.3	126	44.5	9.8	n.s.
BMI [kg/m²]	11	27.5	5.6	125	24.7	3.5	n.s.
work ability index	11	7.2	1.3	125	7.3	1.9	n.s.
cognitive irritation	11	12.8	4	126	11.4	4.8	n.s.
emotional irritation	11	13.5	5.3	126	14.5	5.9	n.s.
PSS sum score	11	5.1	1.8	126	5.3	2.9	n.s.
PHQ sum score	11	2.3	1.9	126	2.7	2.3	n.s.

Jarczok MN, Buckley T, Guendel HO, Boeckelmann I, Mauss D, Thayer JF and Balint EM (2021) 24 h-Heart Rate Variability as a Communication Tool for a Personalized Psychosomatic Consultation in Occupational Health. Front. Neurosci. 15:600865. doi: 10.3389/fnins.2021.600865 2021-01
